# Supplementary material for: The complex genetic landscape of familial MDS and AML reveals pathogenic germline variants
Source: Nat Commun. 2020 Feb 25;11:1044. doi: 10.1038/s41467-020-14829-5 (PMC7042299; doi:10.1038/s41467-020-14829-5)
Supplement: Supplementary file 1 — Supplementary Information [file 41467_2020_14829_MOESM1_ESM.pdf]

Supplementary Information for:

The complex genetic landscape of familial MDS/AML reveals pathogenic germline variants associated with the disease

Rio-Machin *et al.*

Supplementary Figure 1:

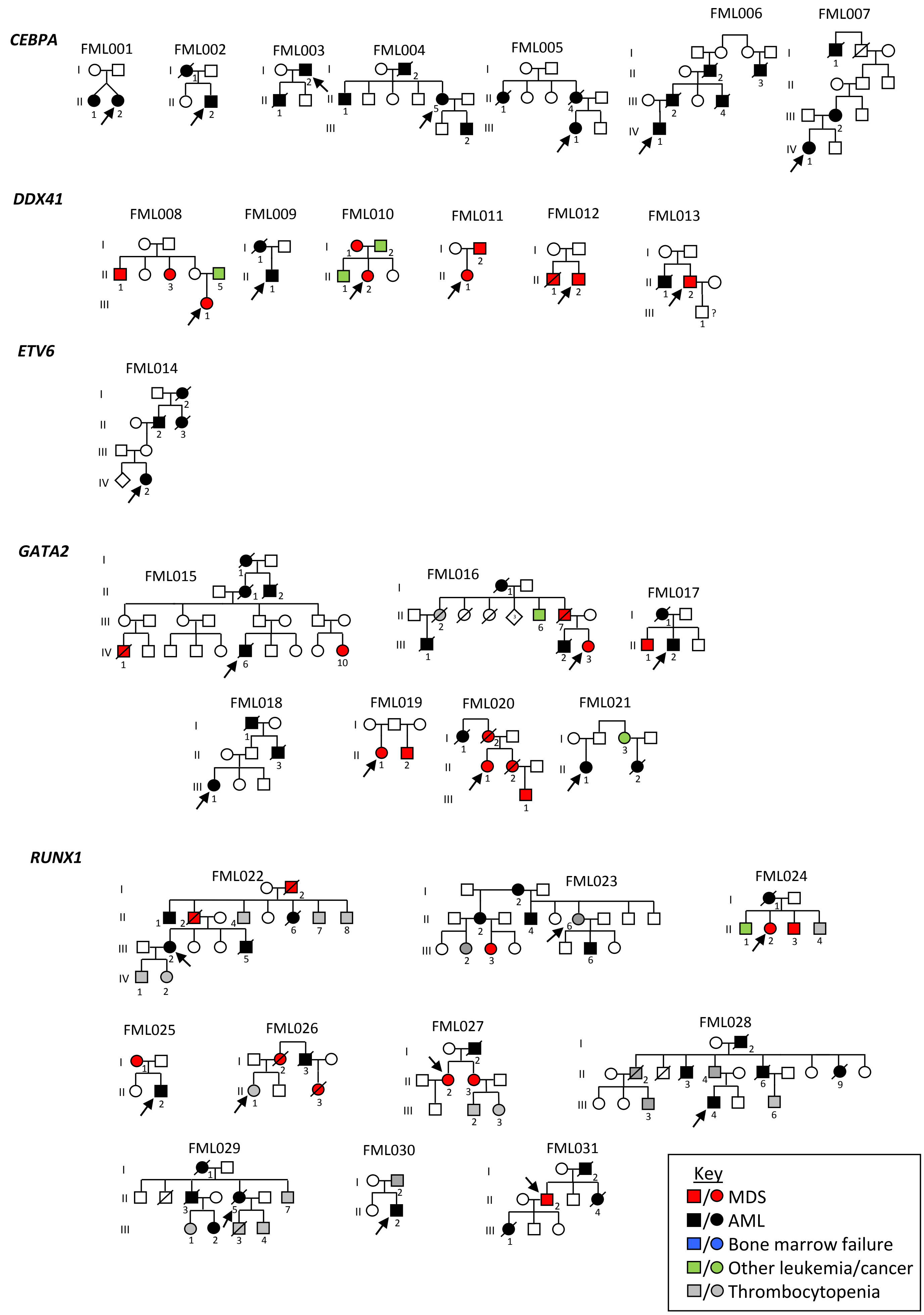

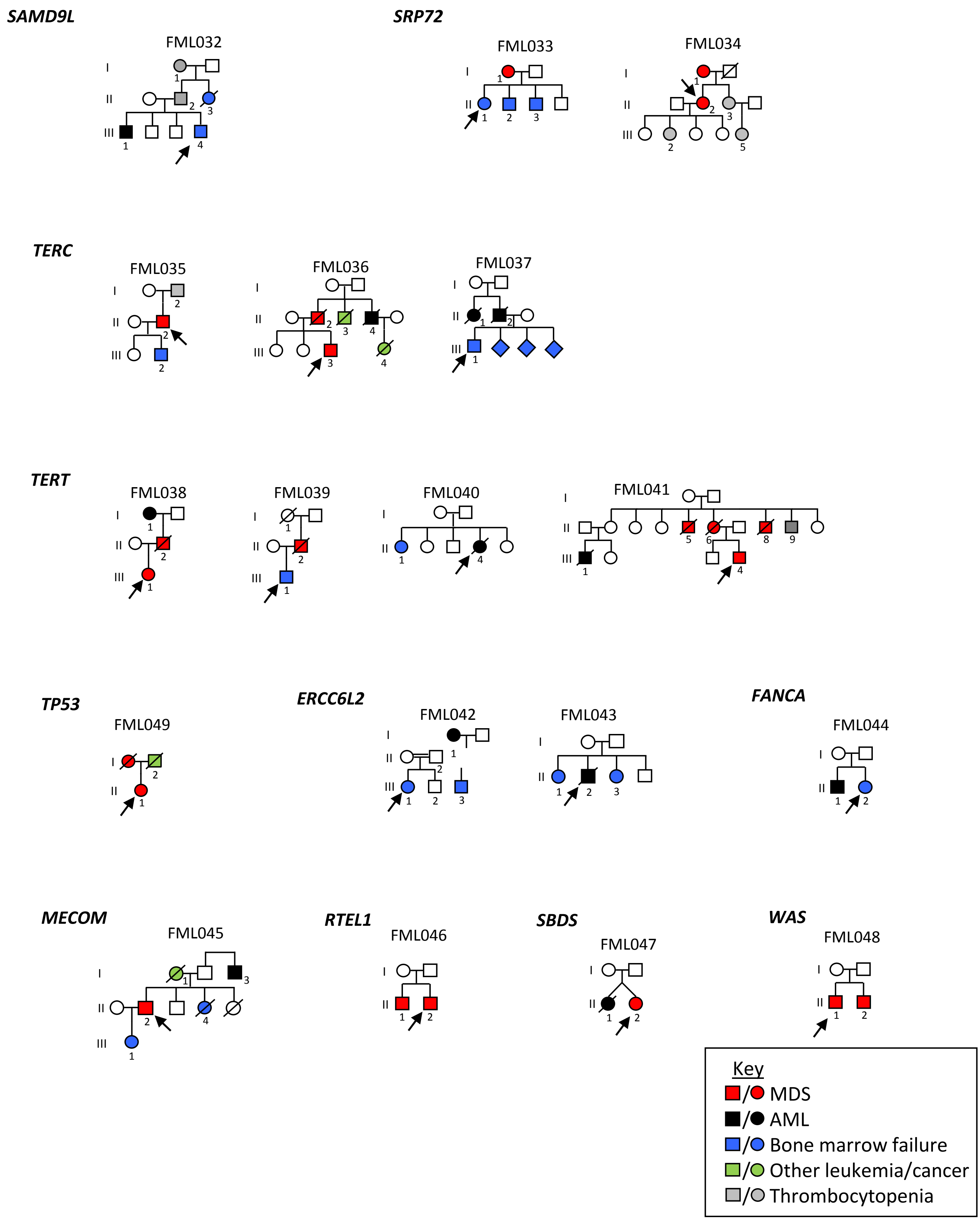

Supplementary Figure 1: Group 1 families. Pedigree representation of the 49 Group 1 families, where a variant has been detected either in an locus with high/moderate level of evidence for gene-disease association, or in one that is emerging from basic research or mutated in other inherited hematological syndromes with high risk of MDS/AML (FML001-FML049). Index case of each family is indicated with an arrow.

Supplementary Figure 2:

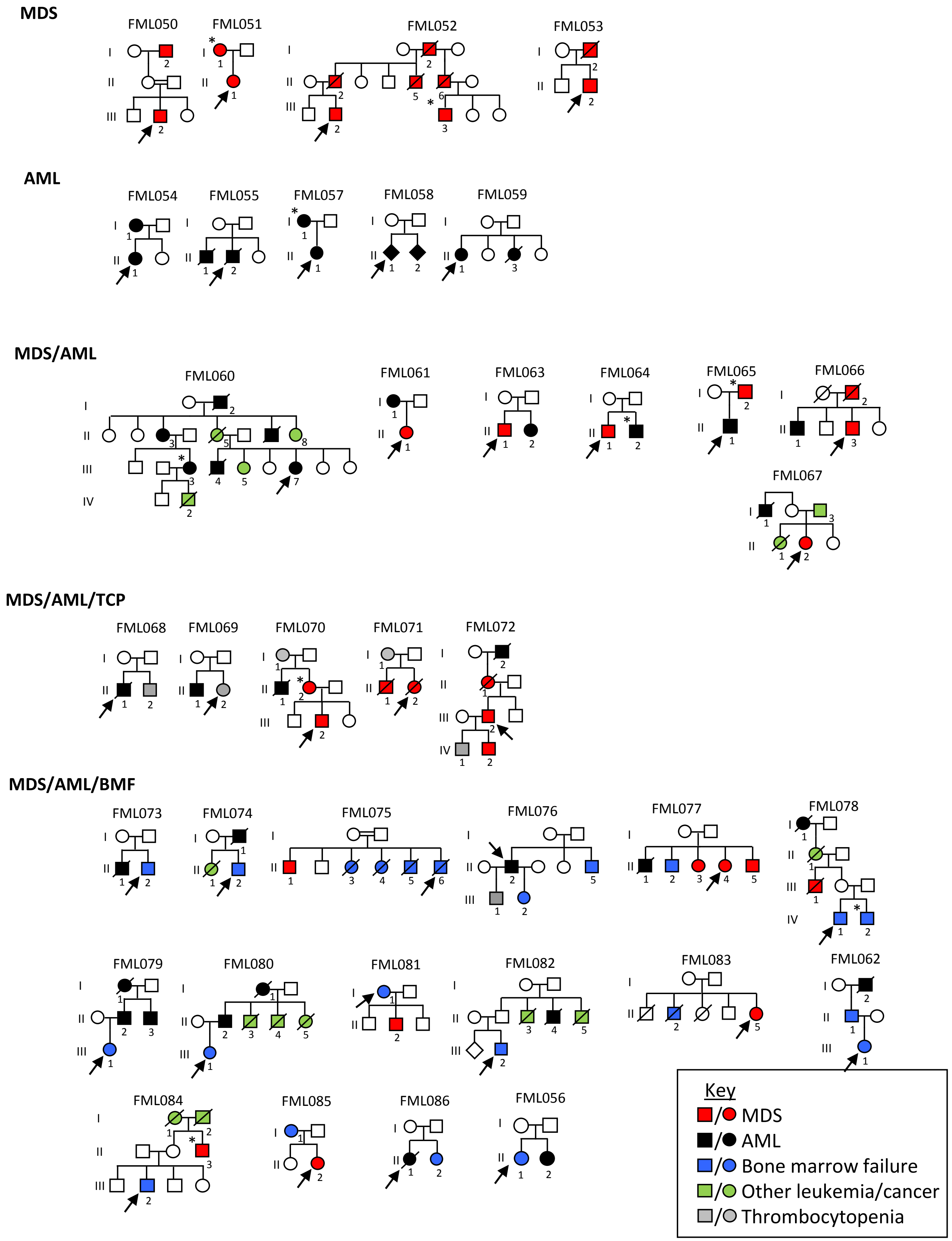

Supplementary Figure 2: Group 2 families. Pedigree representation of the 37 Group 2 families, where the causal genetic lesion has not been defined (FML050-FML086). Index case of each family is indicated with an arrow. Asterisks indicate family members other than the index case where samples were also subjected to WES.

Supplementary Figure 3:

a

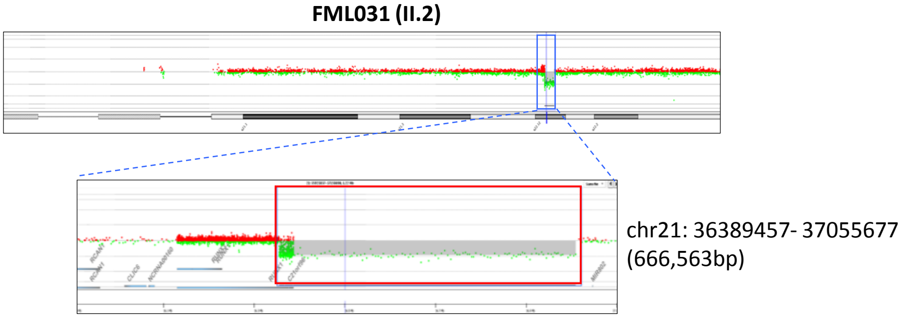

b

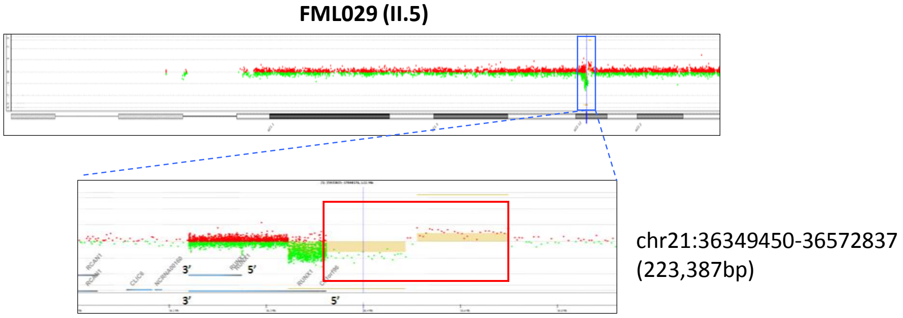

c

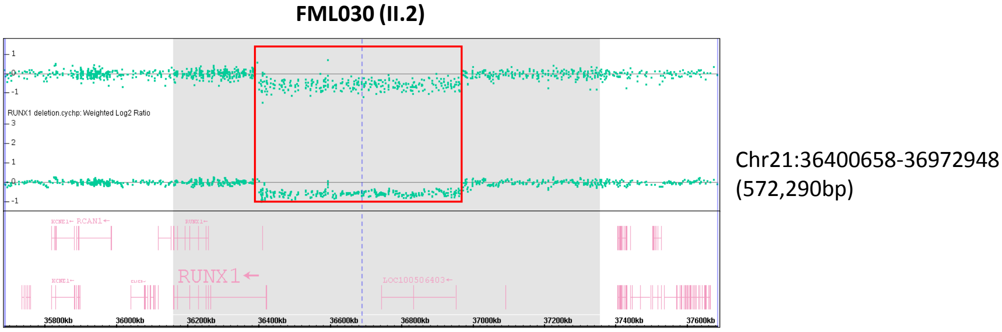

d

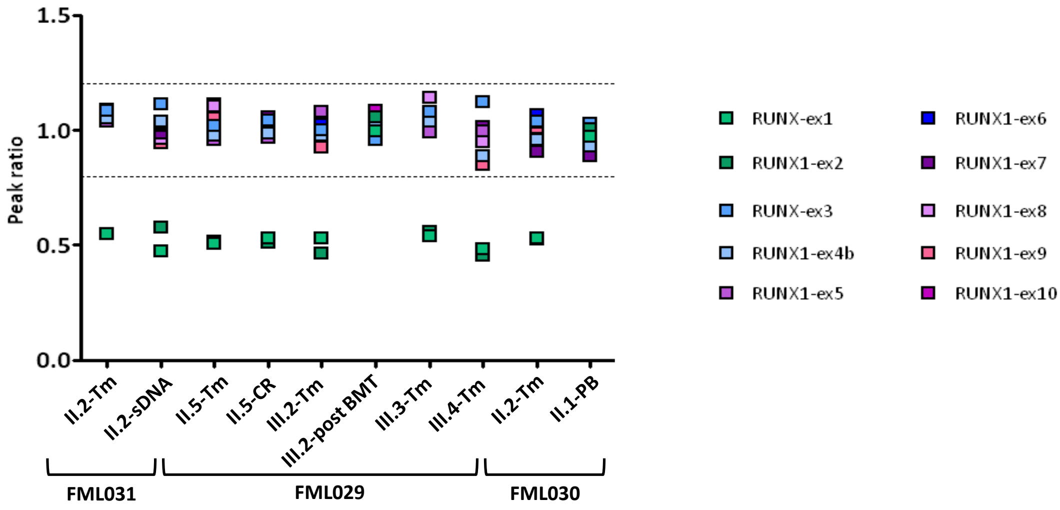

Supplementary Figure 3: *RUNX1* germline deletions. (a) Array CGH analysis of salivary DNA from II.2 of family FML031 revealed a *RUNX1* 666kb deletion (chr21: 36389457- 37055677). **(b)** A *RUNX1* compound duplication (chr21: 36594603- 36768032) and deletion (chr21:36349450-36572837) was identified by Array CGH in patient II.5 of family FML029. **(c)** SNP array in II.2 of family FML030 identified a RUNX1 deletion (chr21: 36400658-36972948). **(d)** MLPA analysis of multiple individuals/samples from pedigrees FML029 (II.5, III.2, III.3 and III.4), FML030 (II.2 and I.1) and FML031 (tumour and salivary DNA from II.2, samples from I.2 were not available) confirmed heterozygous loss of *RUNX1* exons 1 and 2 (shown in green) in affected individuals, with normal peak ratios in remaining exons. (Key: PB-peripheral blood; sDNA-salivary DNA; Tm-tumour; CR- complete remission; post BMT- post allogeneic HSCT).

Supplementary Figure 4:

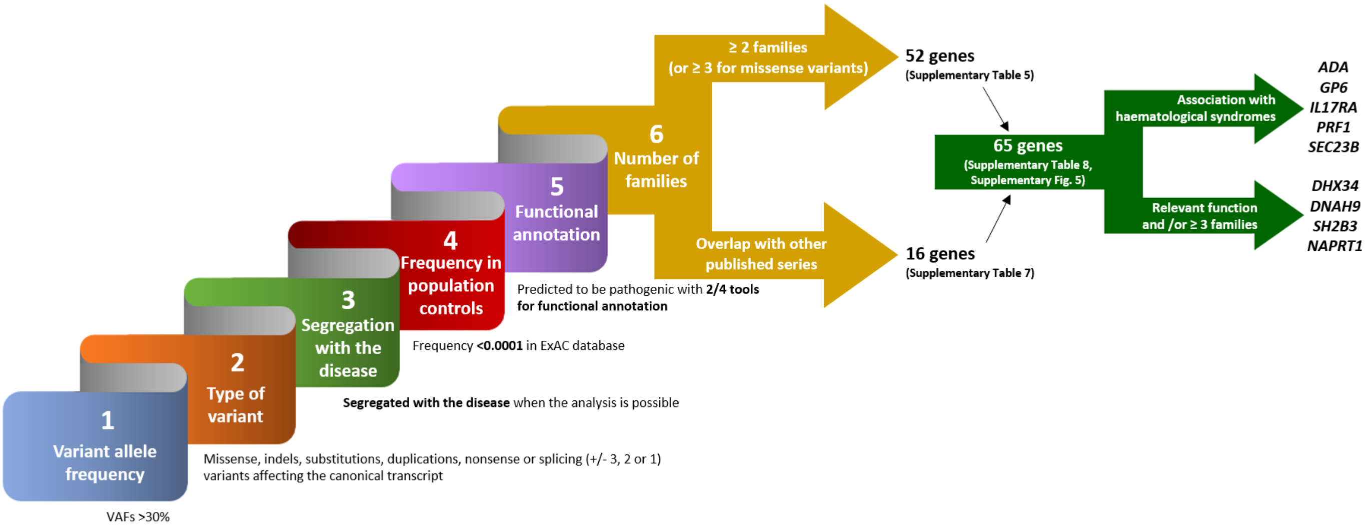

Supplementary Figure 4: Filtering pipeline. Schematic representation of the different steps followed to prioritize the most relevant candidate genes in our series.

Supplementary Figure 5:

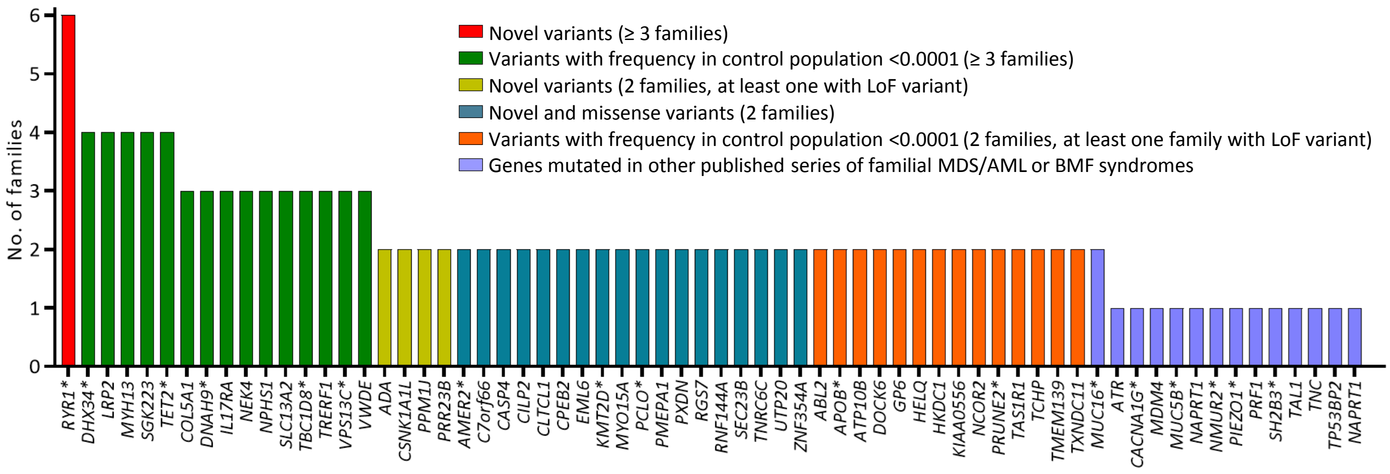

Supplementary Figure 5: Novel candidate loci meeting the selection criteria. Number of families with variants in the 65 novel candidate loci meeting the selection criteria. (*Novel variants:* not present in control population ExAC database; *LoF variants:* frameshift insertions/deletions/substitutions/duplications and nonsense variants; *Missense variants:* missense and nonframeshift/inframe variants). Asterisks indicate genes mutated also in sporadic AML based on The Cancer Genome Atlas AML database[44] and Tyner *et al.* data[45].

Supplementary figure 6:

ADA

FML071 (II.2)
c.259_260insT:p.(Glu88Argfs*34)

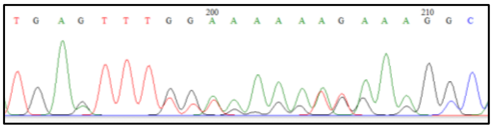

FML056
c.C7T:p.(Gln3*)

II.1

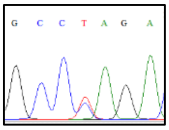

II.2

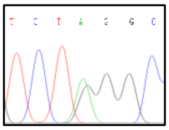

CLTCL1

FML079 (III.1)
c.T4670C:p.(Phe1557Ser)

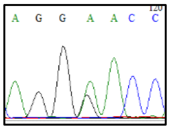

CSNK1A1L

FML071 (II.2)
c.946G>T:p.(Gly316Cys)

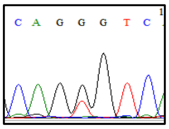

FML065
c.360_361insA:p.(Ile121Asnfs*5)

II.1

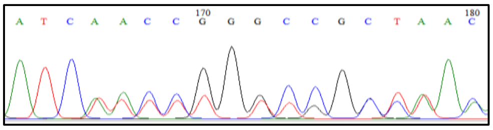

II.2

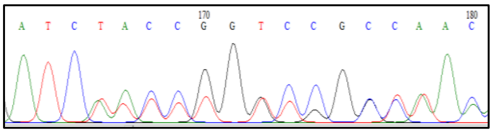

DHX34

FML071 (II.2)
c.1912G>A:p.(Asp638Asn)

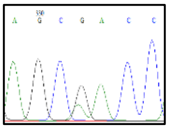

FML061 (II.1)
c.1332G>T:p.(Glu444Asp)

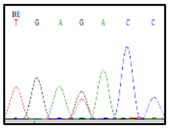

FML065
c.1544A>G:p.(Tyr515Cys)

I.2

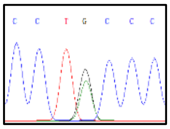

II.1

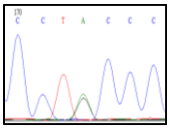

FML055 (II.2)
c.2689C>T:p.(Arg897Cys)

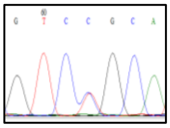

DNAH9

FML055 (II.2)

c.4667A>T:p.(Asp1556Val)

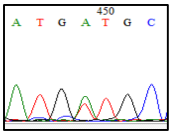

c.4969T>A:p.(Tyr1657Asn)

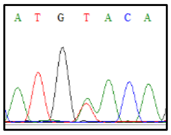

FML056 (II.1)

c.662T>C:p.(Val221Ala)

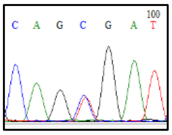

c.6457G>A:p.(Ala2153Thr)

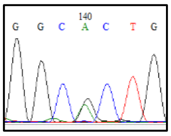

FML059 (II.1)

c.9703G>A:p.(Glu3235Lys)

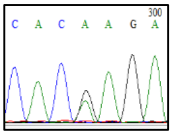

GP6

FML063 (II.1)
c.T1289A:p.(Met430Lys)

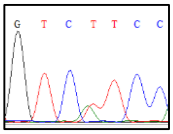

FML055 (II.2)
c.A475T:p.(Arg159*)

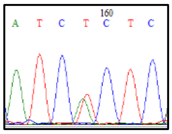

HELQ

FML049 (III.2)
c.3095delA:p.(Tyr1032fs)

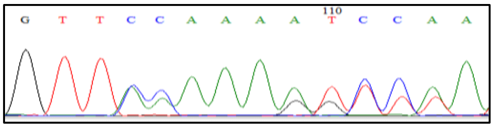

FML071 (II.2)
c.C2063A:p.(Ala688Asp)

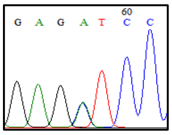

HKDC1

FML061 (II.1)
c.G1831C:p.(Asp611His)

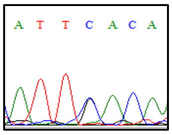

FML083 (II.5)
c.C2650T:p.(Arg884*)

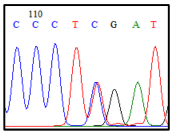

IL17RA

FML053 (II.2)
c.127T>G:p.(Cys43Gly)

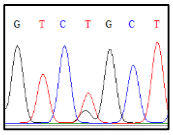

FML068 (II.2)
c.1291C>T:p.(Arg431Cys)

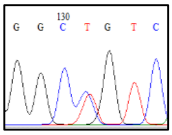

FML049 (III.2)
c.G1403G>A:p.(Arg468His)

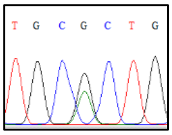

KMT2D

FML050
c.C15053G:p.(Thr5018Arg)

I.1

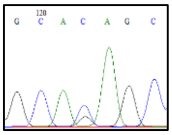

II.1

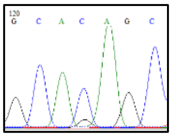

FML076 (II.2)
c.G8291A:p.(Gly2764Glu)

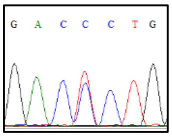

LRP2

FML080 (III.1)
c.2035A>G:p.(Arg679Gly)

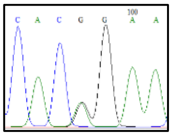

NAPRT1

FML064
c.658_659insGTCACTTCCTTT:p.(Ser220delinsValThrSerPheSer)

II.1

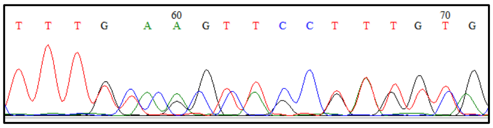

II.2

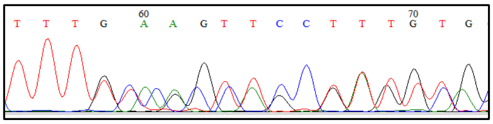

NPHS1

FML068 (II.2)
c.3455C>T:p.(Thr1152Met)

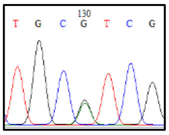

FML055 (II.2)
c.c.614_621delCACCCCGG:p.(Thr205Lysfs*4)

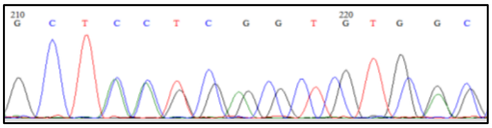

c.614_615insTT:p.(Pro206Tyrfs*30)

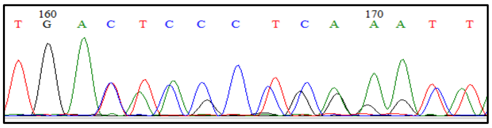

PPM1J

FML071 (II.2)
c.1233C>G:p.(Asp411Glu)

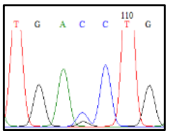

FML083 (II.5)
c.32_33delAC:p.(His11Profs*51)

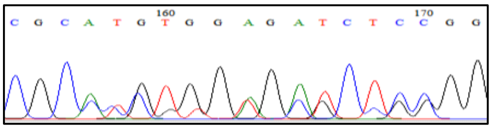

PRR23B

FML069 (II.2)
c.361G>A:p.(Gly121Arg)

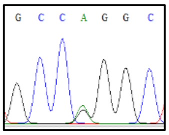

FML083 (II.5)
c.566_567insT:p.(Arg190Profs*27)

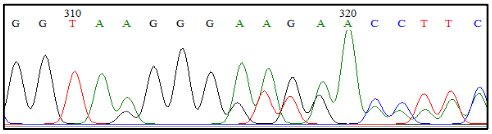

PRUNE2

FML075 (II.6)
c.G2416C:p.(Glu806Gln)

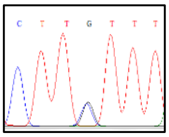

FML074 (II.2)
c.2598delC:p.(Gly866fs)

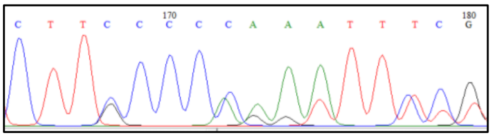

PXDN

FML070
c.C3760T:p.(Pro1254Ser)

II.2

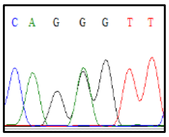

III.2

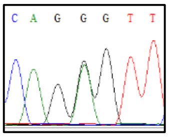

PRF1

FML085 (II.2)
c.G823A:p.Glu275Lys

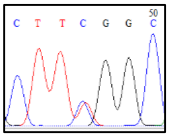

SEC23B

FML061 (II.1)
c.C1625T:p.(Pro542Leu)

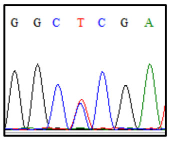

FML062 (III.1)
c.G1083C:p.(Glu361Asp)

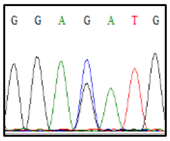

SH2B3

FML085 (II.2)
c.G145A:p.(Ala49Thr)

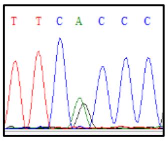

SLC13A2

FML059 (II.1)
c.581C>A:p.(Ser194*)

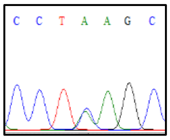

FML080 (III.1)
c.1495C>T:p.(Pro499Ser)

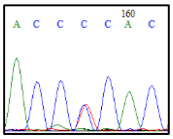

FML081 (I.1)
c.481C>T:p.(ArgR161Cys)

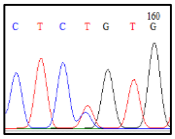

TCHP

FML068 (II.2)
c.A830G:p.(Gln277Arg)

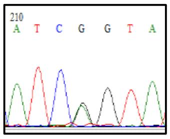

FML079 (III.1)
c.C70T:p.(Arg24*)

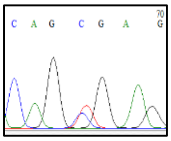

TET2

FML054
c.722C>T:p.(Ala241Val)

I.1

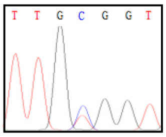

II.1

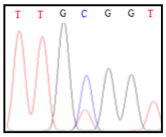

FML081 (I.1)
c.5885C>T:p.(Pro1962Leu)

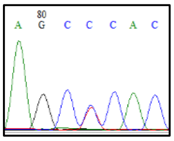

FML075 (II.6)
c.4392C>A:p.(Cys1464*)

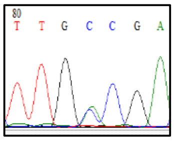

Supplementary Figure 6: Sanger sequencing validation of selected genetic variants. Sanger sequencing traces confirming the presence of 47 variants previously identified by WES (within 22 selected genes), in the corresponding familial samples. Segregation was also validated in all the cases when material from more than one affected individual in the same family was available.

Supplementary Figure 7

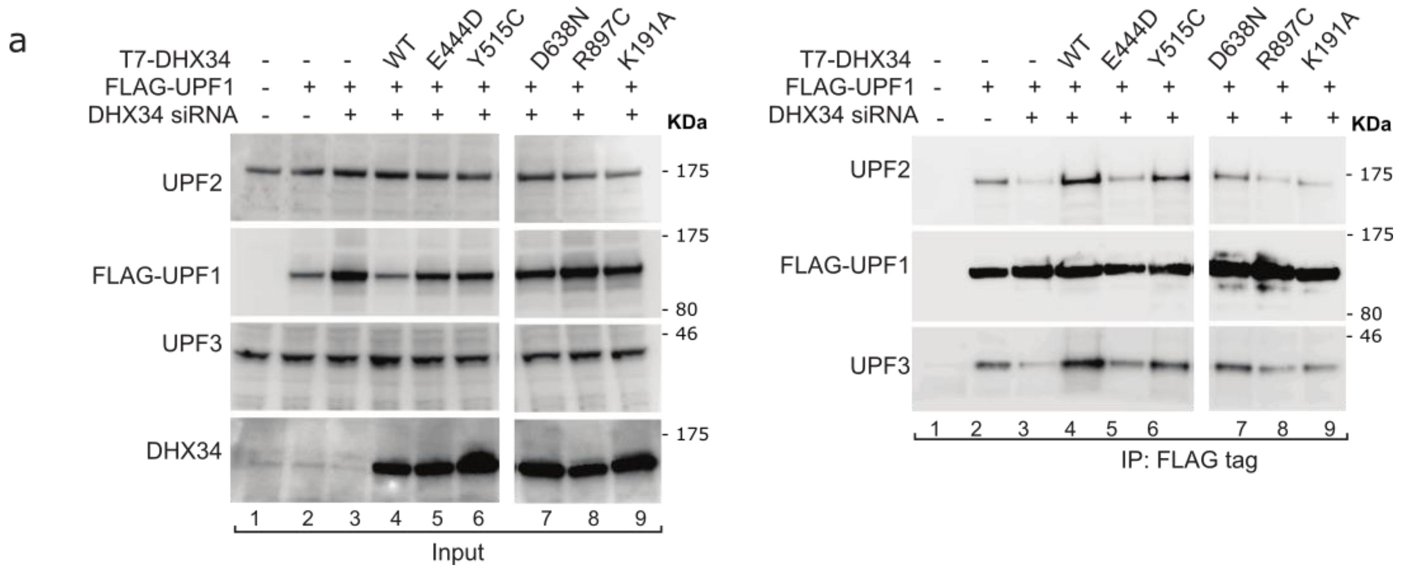

Supplementary Figure 7: *DHX34* variants fail to promote the binding of UPF2 and UPF3 to UPF1. HEK293T cells depleted of *DHX34* with a specific siRNA or transfected with a scrambled non-targeting siRNA (-) were co-transfected with FLAG-UPF1 and a siRNA-resistant wild-type (WT) T7-DHX34 or the indicated *DHX34* variants (with empty vector plasmids as controls (-)). Following anti-FLAG immunoprecipitation, Input (0.5%) and anti-FLAG IPs (20%) were analysed by Western blotting with antibodies for the indicated proteins. Uncropped Western blots are provided as a Source Data file.
