# Supplementary material for: The complex genetic landscape of familial MDS and AML reveals pathogenic germline variants
Source: Nat Commun. 2020 Feb 25;11:1044. doi: 10.1038/s41467-020-14829-5 (PMC7042299; doi:10.1038/s41467-020-14829-5)
Supplement: Supplementary file 3 — Description of Additional Supplementary Files [file 41467_2020_14829_MOESM3_ESM.pdf]

Description of Additional Supplementary Files

File Name: Supplementary Data 1
Description: Summary of the samples included in this study

File Name: Supplementary Data 2
Description: Summary of Group 1 families

File Name: Supplementary Data 3
Description: Summary of Group 2 families

File Name: Supplementary Data 4
Description: Table 3 expanded

File Name: Supplementary Data 5
Description: 52 candidate genes

File Name: Supplementary Data 6
Description: Candidate genes from other series of familial cases

File Name: Supplementary Data 7
Description: Candidate genes common to other series of familial cases

File Name: Supplementary Data 8
Description: Final list of candidate genes

File Name: Supplementary Data 9
Description: Synonymous splicing variants

File Name: Supplementary Data 10
Description: List of primers used for Sanger sequencing validation
